# Supplementary material for: HIVprotI: an integrated web based platform for prediction and design of HIV proteins inhibitors
Source: J Cheminform. 2018 Mar 9;10:12. doi: 10.1186/s13321-018-0266-y (PMC5845081; doi:10.1186/s13321-018-0266-y)
Supplement: Supplementary file 2 — Additional file 2. Source code of HIVProtI web server. [file 13321_2018_266_MOESM2_ESM.zip › HIVprotI_Source-code/hivprotI/weka-3-6-6/doc/weka/associations/tertius/SimpleLinkedList.LinkedListInverseIterator.html]

SimpleLinkedList.LinkedListInverseIterator


---


|  |  |  |
| --- | --- | --- |
| **Overview** \| **Package** \| **Class** \| **Tree** \| **Deprecated** \| **Index** \| **Help** \| **Weka's home** | |  |
| **PREV CLASS**   **NEXT CLASS** | **FRAMES**    **NO FRAMES**     **All Classes** |  |
| SUMMARY: NESTED \| FIELD \| CONSTR \| METHOD | DETAIL: FIELD \| CONSTR \| METHOD |  |


---


## weka.associations.tertius Class SimpleLinkedList.LinkedListInverseIterator

```
java.lang.Object
  weka.associations.tertius.SimpleLinkedList.LinkedListInverseIterator
```

**All Implemented Interfaces:**: java.io.Serializable, RevisionHandler

**Enclosing class:**: SimpleLinkedList

---

``` public class SimpleLinkedList.LinkedListInverseIterator extends java.lang.Object implements java.io.Serializable, RevisionHandler ```

**See Also:**: Serialized Form

---

**Constructor Summary**

`SimpleLinkedList.LinkedListInverseIterator()`


| **Method Summary** | |
| --- | --- |
| `java.lang.String` | `getRevision()`             Returns the revision string. |
| `boolean` | `hasPrevious()` |
| `java.lang.Object` | `previous()` |
| `void` | `remove()` |

**Methods inherited from class java.lang.Object**

`equals, getClass, hashCode, notify, notifyAll, toString, wait, wait, wait`

**Constructor Detail**

### SimpleLinkedList.LinkedListInverseIterator

```
public SimpleLinkedList.LinkedListInverseIterator()
```


**Method Detail**

### hasPrevious

```
public boolean hasPrevious()
```

---


### previous

```
public java.lang.Object previous()
```

---


### remove

```
public void remove()
```

---


### getRevision

```
public java.lang.String getRevision()
```

:   Returns the revision string.

    :   **Specified by:**: `getRevision` in interface `RevisionHandler`
    :   **Returns:**: the revision


---


|  |  |  |
| --- | --- | --- |
| **Overview** \| **Package** \| **Class** \| **Tree** \| **Deprecated** \| **Index** \| **Help** \| **Weka's home** | |  |
| **PREV CLASS**   **NEXT CLASS** | **FRAMES**    **NO FRAMES**     **All Classes** |  |
| SUMMARY: NESTED \| FIELD \| CONSTR \| METHOD | DETAIL: FIELD \| CONSTR \| METHOD |  |


---
